# Patients with dementia syndrome in public and private services in southern Brazil

**DOI:** 10.1590/S1980-57642015DN91000010

**Published:** 2015

**Authors:** Carlos Henrique Ferreira Camargo, Giuliano Retzlaff, Filipe Fernandes Justus, Marcelo Resende

**Affiliations:** 1 MD, PhD; Neurology Service, Medicine Department, University Teaching Hospital – State University of Ponta Grossa, Ponta Grossa, Brazil; 2 Medicine student; Neurology Service, Medicine Department, University Teaching Hospital – State University of Ponta Grossa, Ponta Grossa, Brazil; 3 Medicine student; Neurology Service, Medicine Department, University Teaching Hospital – State University of Ponta Grossa, Ponta Grossa, Brazil; 4 MD; Neurology Service, Medicine Department, University Teaching Hospital – State University of Ponta Grossa, Ponta Grossa, Brazil.

**Keywords:** dementia, epidemiology, Alzheimer's disease, outpatient clinics, public service, private service

## Abstract

Dementia is characterized by deficits in more than one cognitive domain, affecting language, praxis, gnosis, memory or executive functions. Despite the essential economic growth observed in many developing countries, especially over the last century, huge differences remain in health care, whether among nations themselves or across different regions of the same country.

**Objective:**

The aim of this study was to assess the management and main features of dementia, comparing public (PUBL) and private (PRIV) reference services.

**Methods:**

We performed a retrospective analysis of medical records of subjects with dementia. Sociocultural data, mean follow-up time in the service, Mini-mental State Examination (MMSE) scores at admission, main diagnosis of dementia, family history of dementia, comorbidities, imaging methods and treatment were assessed.

**Results:**

the time elapsed before admission in the service of the PUBL group (2.08±2.06 years) was higher than for the PRIV group (1.24±2.55 years) (p=0.0356); the MMSE score at admission in the PUBL group (15.05±8.16 years) was lower than in the PRIV group (18.95±6.69 years) (p=0.016); the PUBL group showed lower treatment coverage with cholinesterase inhibitors (52.94%) than the PRIV group (84.93%) (p=0.0001).

**Conclusion:**

Patients seeking the public health service have less access to medical care, reaching the system at more advanced stages of disease. The public service also offered lower pharmacological coverage.

## Abstract (pt)

Demência é caracterizada por déficts em mais de um domínio cognitivo, afetando linguagem, praxia, gnosia, memória ou função executiva. Apesar do importante crescimento econômico observado em diversos países em desenvolvimento, especialmente no último século, ainda existem diferenças significativas no que se refere aos cuidados em saúde, seja em meio aos países entre si, ou mesmo quando observadas regiões dentro do mesmo país.

**Objetivo:**

Avaliar o manejo e principais características da demência, comparando serviços de referência, exclusivamente público (PUBL) e exclusivamente privado (PRIV).

**Métodos:**

Analise retrospectiva dos prontuários médicos dos indivíduos com demência, sendo observados dados socioculturais, tempo médio até o acompanhamento, escore do Mini-exame do Estado Mental (MEEM) na admissão, diagnóstico etiológico da demência, história familiar demencial, comorbidades, métodos de imagem e tratamento.

**Resultados:**

O tempo até o acompanhamento no grupo PUBL (2,08±2,06 anos) foi maior do que no grupo PRIV (1,24±2,55 anos) (p=0,0356); o escore do MEEM no grupo PUBL (15,05±8,16) foi menor do que no grupo PRIV (18,95± 6,69) (p=0,016); o grupo PUBL apresentou menor cobertura terapêutica (42,94%) com inibidores da acetilcolinesterase do que o grupo PRIV (84,93%) (p=0,0001)

**Conclusão:**

Os pacientes que buscaram o serviço público possuíam menor acesso aos cuidados de saúde, chegando ao sistema em estágios mais avançados da doença, apresentando também menor cobertura farmacológica.

## INTRODUCTION

There are numerous factors for people seeking health care, such as limited activity, number of chronic diseases, severity of symptoms and self-reported health status.^1^ These reasons are much more common among the elderly, especially in those suffering from dementia.^2^

Dementia is characterized by deficits in more than one cognitive domain, affecting language, praxis, gnosis, memory or executive functions.^3,4^ Memory must be affected, although it may remain relatively preserved in initial stages of some forms of dementia.^5^ Also, the condition must be sufficiently severe to interfere in the patient's daily activities.^4,5^

Epidemiological studies have demonstrated that dementia incidence and prevalence has increased exponentially with the advance of age.^4^ Despite scarcity of epidemiological surveys in Brazil, it is known that progressive population aging imposes great burden to society.^6^ This economic, psychological and social burden is set to increase, considering estimates that 25% of Brazilians will be elderly by 2050.6 In developed countries, dementia prevalence doubles every 5 years.^7^

Among the different causes of dementia, the four most common diseases are Alzheimer's disease (AD), vascular dementia (VD), frontotemporal dementia (FTD) and Lewy's Bodies dementia (LD).^5,6^ AD figures as the most frequent form of dementia, representing 50 to 80% of all cases.^6^ FTD is responsible for 5 to 10% of dementia cases.^6^ There is also Parkinson's disease with dementia (PDD),^8^ another significant cause of dementia, sharing several features with LD, complicating their specific diagnoses,^9^ plus mixed dementia (MD), which exhibits clinical and pathophysiological findings of both AD and VD concomitantly.^10^

Despite the essential economic growth observed in many developing countries, especially over the last century, huge differences remain in health care, whether among nations themselves or across different regions of the same country. This can be explained, in most cases, by unequal economic development, insufficient distribution of financial resources, inefficiency of public health care programs or because of differences between the health care services utilized.^11^

In Brazil, since 1989, public healthcare has been based on the *Sistema Único de Saúde - SUS* (Unified Health System), which grants free health care at all levels of complexity to all citizens, respecting their individual needs, aiming to prioritize the most critical cases.^12^ The system is financed by taxes and social contributions, but private initiative also plays a part in the process by providing physical infrastructure and human resources. Numerous clinics, hospitals and laboratories, as well as many health professionals, engage in activities for public and private services concomitantly.^13^ The SUS has financing and management issues in several regions of the country; this creates significant disparities between public and private healthcare systems, especially with regard to patient accommodation, health care and resource availability, and individual follow-up.^14^

In view of the scenario outlined, the aim of this study was to assess the management and main features of dementia, comparing public and private referral services in the city of Ponta Grossa, southern Brazil.

## METHODS

A retrospective analysis of the medical records of all patients admitted by the SUS in the Neurology outpatient unit of the University Teaching Hospital of Campos Gerais (HURCG) and also of all patients treated at the Neurology service of the private clinic *Inovare Serviços de Saúde Ltda* (INOVARE), spanning from the beginning of 2011 to the end of 2013. Both services were located in the city of Ponta Grossa, state of Paraná, southern region of Brazil. Attending neurologists were the same, applying the assessment and treatment criteria for dementia determined by the American Academy of Neurology and the Brazilian Academy of Neurology.^15,16^

This study was approved by the Research Ethics Committee of the State University of Ponta Grossa, Brazil (process no. 16132 - 2012).

**Selection criteria.** Medical records with the following characteristics were included:

[1] presence of sufficient data to characterize the DS and to determine its specific etiological entity; and
[2] patients followed by a physician working at both the services evaluated.

**Data collection.** Clinical records were systematically assessed. Demographic and sociocultural data were collected: age, gender, ethnicity, schooling, occupation, residence, smoking and alcohol abuse, along with clinical data: time elapsed since the onset of symptoms and admission in the neurology service, score on the Mini-mental State Examination (MMSE)^17^ at admission, family history of dementia, comorbidities, brain imaging tests required and the type of therapeutic approach for dementia treatment.

After data collection, patients with dementia were classified into specific diagnoses according to established diagnostic criteria: the DSM-IV criteria^3^ were used to confirm the diagnosis of AD, VD and MD; for confirmation of PDD, the Diagnostic Criteria for Mild Cognitive Impairment in Parkinson's Disease were used;^18^ for LD, the criteria in the Consensus for Clinical and Pathological Diagnosis of Dementia with Lewy's Bodies were employed;^19^ and for the diagnosis of FTD, the criteria of the Consensus for Clinical and Pathological Diagnosis of Frontotemporal Dementia were applied.^20^

For AD, based on individual scores on the MMSE^14^ at admission, patients were staged according to the severity of the dementia. Scores below 10 defined advanced disease, and above 19 to 26 defined mild disease.^21^

**Comparison between groups.** Subjects fulfilling the selection criteria were divided into two groups: the PUBL group, based on the medical records of patients attended at the HURCG, representing the public healthcare system; and the PRIV group, based on the medical records assessed at INOVARE, representing the private healthcare system.

**Statistics.** All data were tested according to the distribution pattern (normal or non-normal). Statistical differences between group means were determined using the one-tailed Student's t-test for normal distributions, and the Mann-Whitney test for non-normal distributions. For the differences between the expected values and the values actually found, the Chi-square test with Yates correction and Fisher's exact test were used. The results were expressed as mean ± s.d. (standard deviation). Differences were considered significant for p<0.05.

## RESULTS

Thirty-four patients for the PUBL group and 166 patients for the PRIV group were selected. Subjects treated in the private service had higher mean age and schooling and also lived more frequently in urban areas and had lower rates of smoking and alcohol abuse when compared to the patients that sought the public service (Table 1). The gender proportion (male:female) differed significantly between group, with 0.48:1 in the PUBL group versus 1:2.13 in the PRIV group (p=0.0002).

**Table 1:** Epidemiological and sociocultural data of patients with dementia.

	Publ group	Priv group	p
Mean age (years)	75.08±7.43	80.55±8.13	0.0001
Male:female ratio	0.48:1	1:2.13	0.0002
Mean schooling years	4.82±3.23	8.60±4.41	< 0.001
Urban residents	26 (76.48%)	158 (95.19%)	0.0015
Do not work	34 (100%)	159 (95.79%)	0.6051
Smokers	08 (23.52%)	14 (8.43%)	0.0169
Alcoholics	07 (20.58%)	10 (6.02%)	0.0122

For mean waiting period until admission and mean score on the MMSE at admission, a difference was observed between groups with regard to the specific diagnosis for dementia (Tables 2 and 3). The patients under the public healthcare system took longer to be seen by the specialist, waiting on average 2.08 (±2.06) years, versus 1.24 (±2.55) years in the private service (p=0.0356). Patients from the PRIV group had higher scores on the MMSE at admission, with an average of 18.95 (±6.69) versus 15.05 (±8.16) in the PUBL group (p=0.0016).

**Table 2:** Mean years elapsed until treatment.

	Publ group	Priv group	p
All patients	2.08± 2.06	1.24±2.55	0.0356
Alzheimer’s disease	2.44±1.88	1.32±2.35	0.0293
Vascular disease	0.83±2.22	0.70±2.71	0.4603
Lewy’s Bodies dementia	2±2.64	1.82±2.76	0.4598
Frontotemporal dementia	–	2.50±3.53	–
Parkinson’s disease with dementia	2.66±1.52	–0.55±4.12	0.1320
Mixed dementia	3±4.24	1.14±1.71	0.1014

**Table 3:** Mean MMSE* scores at time of admission to service.

	Publ group	Priv group	p
All patients	15.05±8.16	18.95±6.69	0.0016
Alzheimer’s disease	14±6.52	18.73±6.71	0.0032
Vascular disease	9.5±6.12	22.9±5.13	0.0001
Lewy’s Bodies dementia	23.66±2.51	19.23±6.21	0.1235
Frontotemporal dementia	28	28.00±1.41	–
Parkinson’s disease with dementia	23.66±4.93	17.66±7.93	0.1269
Mixed dementia	16.5±13.43	18.42±5.67	0.3407

* Mini-mental State Exam.^17^

Concerning investigation of the dementia, there was significant difference regarding the standard brain imaging method for diagnostic support. In the PUBL group, 29 (85.29%) individuals underwent cranial computed tomography (CCT) as the exam of choice, while in the PRIV group, only 66 (39.75%) had this test (p=0.0001). This pattern was inverted for head magnetic resonance imaging (HMRI), where 98 (59.03%) patients were submitted to the exam in the PRIV group, versus only 8 (23.52%) patients in the PUBL group (p=0.0002).

Pharmacological management through acetylcholinesterase inhibitors was substantially higher in patients treated under the private system, totaling 141 (84.93%) subjects, compared with only 18 (52.94%) subjects in the public system (p=0.0001). It was observed that 9 (26.47%) individuals in the PUBL group used memantine, versus only 12 (7.22%) in the PRIV group (p=0.0028).

**Figure 1:**
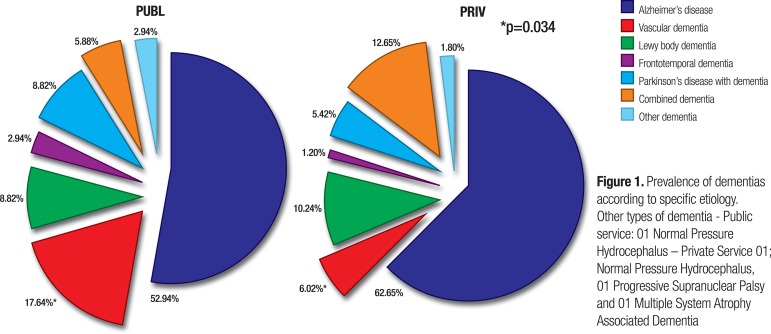
Prevalence of dementias according to specific etiology. Other types of dementia - Public service: 01 Normal Pressure Hydrocephalus – Private Service 01; Normal Pressure Hydrocephalus, 01 Progressive Supranuclear Palsy and 01 Multiple System Atrophy Associated Dementia

There was no statistically significant difference between groups for distribution of dementia into specific diagnoses, except for VD, which was more common among patients seeking the public healthcare system (p=0.0346) (Figure 1). Risk factors associated with vascular disease (systemic arterial hypertension, diabetes, dyslipidemia, alcohol abuse, personal history of stroke and cardiovascular disease) were similarly present in both groups, except for smoking. Smoking was absent in the PRIV group, while in PUBL group, 4 (66.66%) patients were smokers (p=0.0082).

For AD, regarding severity of the disease when seen by the neurologist, relevant statistical disparities between the groups were found (Table 4). In AD, the PRIV group had 52 (50%) patients admitted in the service at mild stage of disease, while in the PUBL group, only 3 (16.66%) patients with this stage were admitted (p=0.01). Among subjects from the private system, only 12 (11.53%) individuals had dementia at advanced stage upon seeking the service, while in the public system 7 (38.88%) patients with advanced stage dementia were admitted (p=0.0081). The pharmacological therapeutic approach used in patients with AD showed significant differences between groups with mild dementia, as depicted in Table 5.

**Table 4:** Clinical features of patients with Alzheimer’s disease.

	Publ group	Priv group	p
Mean age (years)	76.27 (±6.76)	80.70 (±7.67)	0.0117
Mean schooling years	6.00 (±2.82)	8.74 (±4.45)	0.0065
Time elapsed until treatment (years)	2.44 (±1.88)	1.32 (±2.35)	0.0293
MMSE score on admission	14 (±6.52)	18.73 (±6.71)	0.0032
HMRI as standard imaging exam	4 (22.22%)	62 (59.61%)	0.0044
CCT scan as standard imaging exam	15 (83.33%)	37 (35.57%)	0.0167

MMSE: Mini-mental State Exam;^17^ HMRI: Head Magnetic Resonance Imaging; CCT: Cranial Computed Tomography.

**Table 5:** Pharmacological therapeutic approach in mild, moderate and severe Alzheimer’s disease (AD), by number of patients.

		Publ group	Priv group	p
AD (all patients)	IAch * use	11 (61.11%)	91 (87.75%)	0.0113
	Memantine use	5 (27.77%)	11 (10.57%)	0.1566
	No pharmacological approach	5 (27.77%)	9 (8.65%)	0.0340
Mild AD	IAch* use	1 (33.33%)	50 (96.15%)	0.0118
	Memantine use	0 (0%)	0 (0%)	1
	No pharmacological approach	2 (66.66%)	2 (03.85%)	0.0118
Moderate AD	IAch* use	5 (62.5%)	34 (85%)	0.1589
	Memantine use	0 (0%)	4 (10%)	1
	No pharmacological approach	3 (37.5%)	4 (10%)	0.0795
Severe AD	IAch* use	5 (71.42%)	8 (66.66%)	1
	Memantine use	5 (71.42%)	5 (41.66%)	0.3498
	No pharmacological approach	0 (0%)	0 (0%)	1

* Acetylcholinesterase inhibitors.

## DISCUSSION

Epidemiological and clinical data obtained through group analysis revealed substantial inequalities between those populations treated under the public and private services, despite the fact that all patients were assisted by the same physicians, with similar assessment and therapeutic protocols. The mere finding that the number of subjects selected under the public system represents only about 1/5 of those under the private system could be an important indicator of the obstacles faced by patients with dementia gaining access to an attending neurologist in the public healthcare system. This finding could also be related to a higher number of individuals from this area of the country seeking treatment in the public health system, representing 23.52% of the PUBL Group. Likewise, the lower average years of schooling in this population might have hindered the perception of the signs and symptoms of dementia, perhaps explaining the delay in seeking medical care. Moreover, considering the large socioeconomic difference observed in the population studied (Gini index=0.36 to 0.40 - IBGE/2014), the involvement of cultural and socioeconomic features in the evolution of dementia could be implicated as a contributing factor to this difference. Another relevant issue is the disparity between the physical infrastructure of public and private services, and its influence on the diagnostic and therapeutic management of dementia, revealing the wide inequality in care between different regions of the same country.^11^

In countries where there is lower distribution of economic resources for healthcare, the late diagnosis of dementia could be explained by several elements, such as low schooling of the population, concentration of diagnostic facilities in more remote large urban centers, costs related to diagnostic management and the social stigma itself which accompanies dementia.^22^ It was observed that elderly patients with higher schooling and access to the private service (PRIV group) received better health care, corroborating the findings reported in India^2^ and Cuba,^23^ countries with a high percentage of people living below the poverty line.

In Brazil, besides major social inequality, there are substantial differences in the quality of public healthcare assistance offered across the numerous regions of the country.^2^ In a study performed by Dias et al.^24^ in Brazil's southeast region, subjects assessed under the public system had lower waiting times before being seen (22.6 months) and slightly higher mean MMSE scores (16.4±5.0) compared to the present study, probably because of the better quality of health care services in this particular region. Another study, by Miranda et al.,^25^ also performed in the southeast region, involving a group of patients with features similar to those of the PUBL group (mean age of 77.8±6.^8^ years, mean schooling of 3 years), showed an even shorter waiting time until diagnosis (1.5 years). This difference is more evident when comparing to the reality of developed countries. In a German study, conducted by Froelich et al.,^26^ the research subjects had a mean MMSE score of 19.7 points and mean waiting time until follow-up of 15.8 months, reflecting the higher schooling and better accessibility to health care in the German population.^24^ On the other hand, these findings for the German public healthcare system resembled the results found in the Brazilian private healthcare system (PRIV group), demonstrating the differences between this two countries' health systems and how much needs to be improved regarding public health care in Brazil to attain the quality and availability of developed countries.

The mean age of the PRIV group was significantly higher, may be explained by elements such as lower incidence of risk factors among these individuals,^27^ higher prevalence of subjects at the mild stage of AD,^28^ higher schooling (providing protection against early manifestation of dementia),^27^ greater concentration in the urban area^18^ and access to the private assistance setting.^2^ In spite of the disparities between gender proportions, both groups presented female predominance, thus confirming this gender as the most frequently affected by dementia.^4,29^ However, this differs from the reality found in some developing countries, where male gender predominates among patients with dementia.^23^

In terms of specific dementia causes, in the PUBL group, the distribution followed the most common epidemiological pattern found in the literature, showing AD as the main cause of SD, followed by VD.^6,29^ However, the PRIV group exhibited a different pattern, with MD as the second-most prevalent, similar to results found in other studies^10,23^. Rockwood et al.,^10^ in Canada, analyzed a sample of 603 patients with dementia, 372 of whom had AD as the etiological diagnosis, 76 MD, 73 VD and 82 with other types of dementia. In a study performed in Havana by Libre et al.,^23^ among 1499 patients with suspected dementia, 46.4% had AD as the underlying disease, followed by MD, responsible for 28.2% of the cases.

Regarding the standard diagnostic approach for ordering brain imaging exams, a notable difference was observed between the systems. HMRI, despite being recommended as the method of choice for diagnostic brain imaging in dementia assessment,^30^ was significantly less available for diagnostic complementation in patients from the PUBL group. Nevertheless, the vast majority of subjects treated under the public system had access to the CCT scan, an acceptable method to complement diagnosis and the therapeutic approach when HMRI is unavailable.^30^

With respect to the therapeutic approach, the use of acetylcholinesterase inhibitors, drugs indicated for the treatment of all stages of AD dementia^26^ and available free of charge under the SUS, differed substantially between the groups. These drugs were more frequently used in patients from the PRIV group. Memantine, a medication predominantly indicated for severe stages of dementia^26^ and unavailable under the SUS, was significantly more used among patients from the PUBL group, indicating the higher number of individuals with advanced cases in this group. In a South African study published by Truter,^31^ it was found that, among prescriptions for the treatment of AD, 24.70% of patients received memantine as a standard pharmacological intervention. This number was similar to the findings of the PUBL group (27.77%), suggesting a more severe clinical profile of patients treated in these services, with probable late diagnosis of AD.

Independently of dementia type, disease stage, or complementary method by which the diagnosis was obtained, patients from both groups had good conditions of drug administration and continuous follow-up. Except for the individuals with mild AD under treatment in the public healthcare system, all patients, irrespective of group or disease stage, had therapeutic coverage of over 60% in the use of acetylcholinesterase inhibitors. In a French study performed by Cantegreil-Kallen et al.,^32^ 631 questionnaires answered by general physicians about their patients with AD were analyzed. It was observed that only 50% of patients received prescriptions of acetylcholinesterase inhibitors. Therapeutic coverage with antidementia drugs is even lower in the majority of European countries, probably because of cultural preference for seeing a general physician instead of a specialist for follow-up.^33^

In Brazil, as in other developing countries, there are major shortcomings in the implementation of programs related to elderly welfare and the raising of adequate financial resources to fund high social impact initiatives for the population with dementia, as well as in the effective training of professionals and caregivers for scientifically accurate and humanized care of this patient group.^34^ The social and financial impact related to the care of patients with dementia falls largely on patients' families,^34^ favoring those who have access to private means of care.

Therefore, it can be concluded that patients with dementia seeking the public service in the region analyzed have lower access to health care, entering the system at more severe stages of disease while also having poorer therapeutic coverage in the use of acetylcholinesterase inhibitors compared to patients admitted into the private healthcare system.

## References

[1] Fernández-Olano C, Hidalgo JD, Cerdá-Díaz R (2006). Factors associated with health care utilization by the elderly in
a public health care system. Health Policy.

[2] Channon AA, Andrade MV, Noronha K, Leone T, Dilip TR (2012). Impatient care of the elderly in Brazil and India: Assessing
social inequalities. Soc Sci Med.

[3] American Psychiatry Association (1994). Diagnostic and Statistical Manual of Mental Disorders.

[4] Prince M, Bryce R, Albanese E, Wimo A, Ribeiro W, Ferri CP (2013). The global prevalence of dementia: A systematic review and
metaanalysis. Alzheimers Dement.

[5] Caramelli P, Barbosa MT (2002). Como diagnosticar as quatro causas mais frequentes de
demência. Rev Bras Psiquiatr.

[6] Abbott A (2011). Dementia: a problem for our age. Nature.

[7] Russ TC, Batty GD, Hearnshaw GF, Fenton C, Starr JM (2012). Geographical variation in dementia: systematic review with
meta-analysis. Int J Epidemiol.

[8] Verbaan D, Jeukens-Visser M, Van Laar T (2011). SCOPA - Cognition Cutoof Value for Detection of Parkinson's
Disease Dementia. Mov Disord.

[9] Dodel R, Csoti I, Ebersbach G (2008). Lewy body dementia and Parkinson's disease with
dementia. J Neurol.

[10] Rockwood K, Macknight C, Wentzel C (2006). The Diagnosis of "Mixed" Dementia in the Consortium for the
Investigation of Vascular Impairment of Cognition (CIVIC). Ann N Y Acad Sci.

[11] Fang P, Dong S, Xiao J, Liu C, Feng X, Wang Y (2010). Regional inequality in health and its determinants: Evidence from
China. Health Policy.

[12] Brasil (1988). Constituição (1988). Constituição da
República Federativa do Brasil.

[13] Victora CG, Barreto ML, do Carmo Leal M (2012). Health conditions and health-policy innovations in Brazil: the
way forward. Lancet.

[14] Schiozer RF, Saito CC, Saito R (2011). Financial health and customer satisfaction in private health care
providers in Brazil. Cad Saude Publica.

[15] (2011). Recomendações para o diagnóstico e
tratamento da doença de Alzheimer e demência vascular:
aspectos gerais. Dement Neuropsychol.

[16] Doody RS, Stevens JC, Beck C (2001). Practice parameter: Management of dementia (an evidence-based
review): Report of the Quality Standards Subcommittee of the American
Academy of Neurology. Neurology.

[17] Bertolucci PH, Brucki SM, Campacci SR, Juliano Y (1994). The Mini-Mental State Examination in a general population: impact
of educational status. Arq Neuropsiquiatr.

[18] Litvan I, Goldman JG, Tröster AI (2012). Diagnostic Criteria for Mild Cognitive Impairment in Parkinson's
Disease: Movment Disorder Society Task Force Guidelines. Mov Disord.

[19] McKeith IG, Galasko D, Kosaka K (1996). Consensus guidelines for the clinical and pathologic diagnosis of
dementia with Lewy bodies (DLB): report of the consortium on DLB
international workshop. Neurology.

[20] Neary D, Snowden JS, Gustafson L (1998). Frontotemporal lobar degeneration: a consensus on clinical
diagnostic criteria. Neurology.

[21] (2007). NCCMH Dementia: Supporting People with Dementia and their Carers in
Health and Social Care.

[22] Maestre GE (2012). Assessing Dementia in Resource-Poor Regions. Curr Neurol Neurosci Rep.

[23] Llibre Jde J, Fernández Y, Marcheco B (2009). Prevalence of Dementia and Alzheimer's Disease in a Havana
Municipality: A Community-Based Study among Elderly
Residents. MEDICC Rev.

[24] Dias FLC, Silva RMFL, Moraes EM, Caramelli P (2013). Perfil clínico e autonômico de pacientes com
doença de Alzheimer e demência mista. Rev Assoc Med Bras.

[25] Miranda LFJR, Matoso RO, Rodrigues MV (2011). Factors influencing possible delay in the diagnosis of
Alzheimer's disease. Findings from a tertiary Public University
Hospital. Dement Neuropsychol.

[26] Froelich L, Gertz HJ, Heun R (2004). Donepezil for Alzheimer's disease in clinical practice - The
Donald Study. A multicenter 24 - week clinical Trial in
Germany. Dement Geriatr Cogn Disord.

[27] Sosa-Ortiz AL, Acosta-Castillo I, Prince MJ (2012). Epidemiology of Dementias and Alzheimer's Disease. Arch Med Res.

[28] Paradise M, Walker Z, Cooper C (2009). Prediction of survival in Alzheimer's disease - The LASER AD
longitudinal study. Int J Geriatr Psychiatry.

[29] Herrera Jr E, Caramelli P, Silveira ASB, Nitrini R (2002). Epidemiologic Survey of Dementia in a Community-Dwelling
Brazilian Population. Alzheimer Dis Assoc Disord.

[30] Engelhardt E, Tocquer C, André C (2011). Demência Vascular Critérios diagnósticos e
exames complementares. Dement Neuropsychol.

[31] Truter I (2010). Prescribing of drugs for Alzhemeir's disease: a South African
database analysis. Int Psychogeriatr.

[32] Cantegreil-Kellen I, Turbelin C, Angel P (2006). Dementia management in France. Health care and support services
in the community. Dementia.

[33] Waldemar G, Phung KT, Burns A (2007). Access to diagnostic evaluation and treatment for dementia in
Europe. Int J Geriatr Psychiatry.

[34] Cieto BB, Valera GG, Soares GB (2014). Dementia care in public health in Brazil and the
world. Dement Neuropsychol.

